# A Comprehensive Selection of Reference Genes for RT-qPCR Analysis in a Predatory Lady Beetle, *Hippodamia convergens* (Coleoptera: Coccinellidae)

**DOI:** 10.1371/journal.pone.0125868

**Published:** 2015-04-27

**Authors:** Huipeng Pan, Xiaowei Yang, Blair D. Siegfried, Xuguo Zhou

**Affiliations:** 1 Department of Entomology, University of Kentucky, Lexington, Kentucky, United States of America; 2 Department of Entomology, Cornell University, Ithaca, New York, United States of America; 3 Department of Entomology, University of Nebraska, Lincoln, Nebraska, United States of America; Naval Research Laboratory, UNITED STATES

## Abstract

Reverse transcriptase-quantitative polymerase chain reaction (RT-qPCR) is a reliable, rapid, and reproducible technique for measuring and evaluating changes in gene expression. To facilitate gene expression studies and obtain more accurate RT-qPCR data, normalization relative to stable reference genes is required. In this study, expression profiles of seven candidate reference genes, including *β-actin* (*Actin*), *elongation factor 1 α* (*EF1A*), *glyceralde hyde-3-phosphate dehydro-genase* (*GAPDH*), *cyclophilins A* (*CypA*), *vacuolar-type H+-ATPase* (*ATPase*), *28S ribosomal RNA* (*28S*), and *18S ribosomal RNA* (*18S*) from *Hippodamia convergens* were investigated. *H*. *convergens* is an abundant predatory species in the New World, and has been widely used as a biological control agent against sap-sucking insect pests, primarily aphids. A total of four analytical methods, *geNorm*, *Normfinder*, *BestKeeper*, and the *ΔCt* method, were employed to evaluate the performance of these seven genes as endogenous controls under diverse experimental conditions. Additionally, *RefFinder*, a comprehensive evaluation platform integrating the four above mentioned algorithms, ranked the overall stability of these candidate genes. A suite of reference genes were specifically recommended for each experimental condition. Among them, *28S*, *EF1A*, and *CypA* were the best reference genes across different development stages; *GAPDH*, *28S*, and *CypA* were most stable in different tissues. *GAPDH* and *CypA* were most stable in female and male adults and photoperiod conditions, *28S* and *EF1A* were most stable under a range of temperatures, *Actin* and *CypA* were most stable under dietary RNAi condition. This work establishes a standardized RT-qPCR analysis in *H*. *convergens*. Additionally, this study lays a foundation for functional genomics research in *H*. *convergens* and sheds light on the ecological risk assessment of RNAi-based biopesticides on this non-target biological control agent.

## Introduction

Reverse transcriptase-quantitative polymerase chain reaction (RT-qPCR) is a rapid, reliable, and reproducible method measuring gene expression during different biological processes [1]. Although RT-qPCR is considered as a major breakthrough in PCR technology, limitations exist, including variation in RNA extraction, reverse transcription, cDNA concentration, normalization, and PCR efficiency [2, 3, 4]. A commonly used technique in RT-qPCR to normalize the gene expression data is to simultaneously measure the expression of an internal reference gene in the same sample [1]. Reference genes, constitutively expressed to maintain cellular function, are the conventional choice for a standardized reference [1], although it is impractical to find a single reference gene expressed at constant levels under all biotic and abiotic conditions [5, 6].

The convergent lady beetle, *Hippodamia convergens* (Coleoptera: Coccinellidae), is a common lady beetle species in the New World. Although *H*. *convergens* larvae and adults are polyphagous, they prey primarily on aphids, including cotton, pea, melon, cabbage, potato, green peach, and corn leaf aphids [7]. As an effective biological control agent, *H*. *convergens* can reduce aphid densities in greenhouses [8, 9]. When aphids are limited, however, *H*. *convergens* feeds on thrips, small insect larvae and eggs, mites, and honeydew secreted by aphids and other sap-sucking insects, as well as plant based foods, such as pollen, nectar, and petals [10]. Nevertheless, convergent lady beetles must consume preys to reproduce [11].

The advent of the Genomics Era provides unprecedented opportunity to develop novel biopesticides with new modes of action to complement existing biological control agents. Most recently, RNA interference (RNAi)-based transgenic technology has been developed and offers a new approach for the management of insect pests [12–14]. For example, Baum and colleagues (2007) developed transgenetic corn plants that resisted the western corn rootworm, *Diabrotica virgifera virgifera*. By reducing translation of vacuolar *H+-ATPase subunit A* in the pest, the plant increased pest mortality and larval stunting and experienced less root damage as a result [12]. Although technical difficulties and regulatory hurdles still exist [15], the commercialization of a new generation of genetically modified crops is likely [16]. One major ecological concern regarding the biosafety of RNAi-based biopesticides and transgenic crops on the ecosystems is their potential effects on non-target organisms, especially biological control agents which play an important role in current pest management practices [17, 18]. Given the nature of RNAi mechanisms, the non-target effects will likely be through the modulation of gene expressions in non-target organisms [19]. Therefore, RT-qPCR will be a major research tool to evaluate potential non-target effects of this new biotechnology. Despite the demonstrated necessity for systematic validation of reference genes in RT-qPCR studies, normalization procedures for biological control agents have not yet received attention. These natural enemies are a major group of non-target organisms that will be exposed to the RNAi-based biopesticides and transgenic crops in the field.

The objective of this study is to evaluate and select appropriate reference genes with stable expression across various biotic and abiotic conditions in *H*. *convergens*. Seven candidate reference genes including *β-actin* (*Actin*), *elongation factor 1 α* (*EF1A*), *glyceralde hyde-3-phosphate dehydro-genase* (*GAPDH*), *cyclophilins A* (*CypA*), *vacuolar-type H* ^*+*^ *-ATPase* (*ATPase*), *28S ribosomal RNA* (*28S*), and *18S ribosomal RNA* (*18S*) from *H*. *convergens* were tested. The stability of these candidate genes was investigated under three biotic (developmental stage, tissue type, and sex) and three abiotic conditions (temperature, photoperiod, and dietary RNAi). As a result, different sets of reference genes were recommended based on the respective experimental conditions.

## Materials and Methods

### Insects

*Hippodamia convergens* (Coleoptera: Coccinellidae) was purchased from a commercial vender in California (High Sierra Ladybugs, http://www.highsierraladybugs.com). *Hippodamia convergens* larvae and adults were maintained in a growth chamber and provisioned with pea aphids, *Acyrthosiphon pisum*, at 23 ± 1°C temperature, 12L: 12D photoperiod, and 50% relative humidity. *A*. *pisum* clones were kindly provided by Dr. John Obrycki (University of Kentucky), and aphids were maintained at 20–28°C on seedlings of fava beans, *Vicia faba* (Fabales, Fabaceae) in a greenhouse.

### Treatments

#### Biotic factors

The different developmental stages including eggs, four larval instars (collected at the first day of each instar), pupae, and adults. Tissues, including head, midgut, and carcass (body with its head and viscera removed) were dissected from the fourth instars. For the sex treatment, one adult female and male were collected separately and placed in 1.5 ml centrifuge tubes, respectively.

#### Abiotic factors

To examine the influence of temperature, third instars were exposed to 10, 22, and 30°C for 3 h. For photoperiod, third instars were exposed to 16:8, 12:12, and 8:16 h light: dark period for 2 d. For the dietary RNAi treatment, neonate first instars were fed with 15% sucrose solution containing chemically synthesized dsRNAs for *ATPase*, which causes mortality in *H*. *convergens* larvae (*ATPase*-dsRNA) (HP Pan unpublished data), water and *β-glucuronidase* dsRNA (GUS-dsRNA) as controls. At the start of the experiment, *H*. *convergens* neonate larvae (< 24 h old) were kept individually in a petri dish. Each neonate was provisioned with a 2 μl droplet containing 1 μl of dsRNA (8μg /μl) and 1 μl of 30% sucrose solution on a daily basis. In 2 d, 16 μg of dsRNA were supplied to each *H*. *convergens* neonate. At day 3, five individuals in each treatment were collected as one sample for the subsequent RT-qPCR analysis.

For the developmental stage, a total of 15 eggs were collected as one biological replicate, while one pupa was collected individually as one replicate. For the remaining developmental stages, and all other biotic and abiotic conditions, five individuals were collected for each treatment. Each experiment was repeated three times. All collected samples were snap frozen in liquid nitrogen and stored at -80°C in 1.5 ml centrifuge tubes for the subsequent total RNA extraction

### RNA extraction and cDNA synthesis

Total RNA was extracted using TRIzol reagent (Invitrogen, Carlsbad, CA) following the manufacturer’s instruction. The whole body homogenates were centrifuged at 12000 *g* for 15 min and then supernatant was transferred to a new 1.5 ml microcentrifuge tube. A volume of 200 μl chloroform was added to the supernatant and then the mixture was incubated at room temperature for 10 min and then centrifuged at 4°C, 12000 *g* for 15 min. After that, the supernatant was transferred to a new 1.5 ml microcentrifuge tube, 400 μl Isopropyl alcohol was added to it, and the mixture precipitated at room temperature for 10 min. Then, the supernatant was discarded after the mixture was centrifuged at 4°C, 12000 *g* for 8 min, and then 1 ml 75% alcohol was added and centrifuged at 4°C, 7500 *g* for 5 min to wash the pellet. Finally, the pellet was air dried for 8 min and then dissolved in 30–80 μl ddH_2_O. DNase treated total RNA was denatured at 75°C for 5 min and immediately chilled on ice. The concentration of RNA was quantified with a NanoDrop 2000c spectrophotometer with the result for eggs (530.1 ± 28.2 ng/μl), first instars (549.3 ± 35.6 ng/μl), second instars (1377.7 ± 164.7 ng/μl), third instars (2387.9 ± 369.6 ng/μl), fouth instars (1376.5 ± 332.0 ng/μl), pupae (733.8 ± 226.1 ng/μl), adults (914.9 ± 377.5 ng/μl), heads (1038.2 ± 78.9 ng/μl), carcasses (3147.9 ± 372.7 ng/μl), and guts (2591.7 ± 1620.0 ng/μl). First-strand cDNA was synthesized from 2.0 μg of total RNA using the M-MLV reverse transcription kit (Invitrogen, Carlsbad, CA) using a random N primer according the manufacturer’s recommendations. The cDNA was diluted 10-fold for the subsequent RT-qPCR studies.

### Reference gene selection and primer design

Seven reference genes were selected (Table 1). Primers for *18S* and *28S* were designed based on the sequences obtained from NCBI (accession number: EU164617 and EU164644, respectively). For the remaining five reference genes, degenerate primers were designed using CODEHOP (http://blocks.fhcrc.org/codehop.html) according to the conserved amino acid residues among Coleopteran (S1 Table). PCR amplifications were performed in 50 μl reactions containing 10 μl 5×PCR Buffer (Mg^2+^ Plus), 1 μl dNTP mix (10 mM of each nucleotide), 5 μl of each primer (10 μM each), and 0.25 μl of Go Taq (5u /μl) (Promega). The PCR parameters were as follows: one cycle of 94°C for 3 min; 35 cycles of 94°C for 30 s, 59°C for 45 s and 72°C for 1 min; a final cycle of 72°C for 10 min. Amplicons of the expected sizes were purified, cloned into the pCR4-TOPO vector (Invitrogen, Carlsbad, CA), and sent out for sequencing (S2 Table). After the identity of these reference genes was confirmed, primers for the subsequent RT-qPCR analysis were designed online, https://www.idtdna.com/Primerquest/Home/Index.

**Table 1:** Primers used for RT-qPCR.

Gene	Primer sequences (5’-3’)	Length (bp)	Efficiently (%)	R^2^	Linear regression equation
*EF1A*	F: AGTGGAAGACGGAGGGGTTT	123	103.0	0.9995	y = -3.2539x+24.008
	R: ATGGTTCAAGGGATGGGCAA				
*GAPDH*	F: GCCAAGGTGATCCATGACAA	80	103.2	0.9994	y = -3.2473x+26.272
	R: GTCTTCTGAGTGGCAGTTGTAG				
*Actin*	F: CTCCAGAATCCAACACGATACC	125	105.0	0.9995	y = -3.2074x+21.506
	R: CAGGGAGAAGATGACCCAAATC				
*CypA*	F: TGAAGAACTGCGATCCGTTG	112	102.1	0.9882	y = -3.2723x+25.387
	R:TCCATCTACGGCAGCAAATTC				
*18S*	F: TGCATGGCCGTTCTTAGTT	103	101.5	0.9991	y = -3.2864x+11.052
	R: GGGCCTTTGAGGATGTCTAAT				
*28S*	F: CTTAGAGTCGGGTTGCTTGAG	100	96.9	0.9995	y = -3.3985x+12.095
	R: CTCACGGTACTTGTTCGCTATC				
*ATPase*	F: AGAAGCTCGCCCAACGTAAGCATT	82	100.9	0.9999	y = -3.3016x+18.029
	R: AAATCGTCCAGTGCCCTCGTGTACTT				

### Reverse transcriptase-quantitative polymerase chain reaction (RT-qPCR)

PCR reaction (20 μl) contained 7 μl of ddH_2_O, 10 μl of 2×SYBR Green MasterMix (BioRad), 1 μl of each specific primer (10 μM), and 1 μl of the first-strand cDNA template. The RT-qPCR program included an initial denaturation for 3 min at 95°C followed by 40 cycles of denaturation at 95°C for 10 s, annealing for 30 s at 55°C, and extension for 30 s at 72°C. For melting curve analysis, a dissociation step cycle (55°C for 10 s, and then 0.5°C for 10 s until 95°C) was added. Reactions were set up in 96-well format Microseal PCR plates (Bio-Rad) in triplicates, and carried out in a MyiQ single Color Real-Time PCR Detection System (BioRad). Existence of a single peak in melting curve analysis was used to confirm gene-specific amplification and rule out non-specific amplification and primer-dimer generation. A standard curve for each primer pair was constructed with serial dilutions of cDNA (1, 1/5, 1/25, 1/125, 1/625, and 1/3125). PCR amplification efficiency (E) was calculated according to the equation: E = (10^[-1/slope]^ -1)×100.

### Expression stability analysis of candidate reference genes

The stability of each candidate reference gene was evaluated by algorithms *geNorm* [1], *NormFinder* [20], *BestKeeper* [21], and the *ΔCt* method [22]. Finally, we compared and ranked the tested candidates based on the comprehensive web-based analysis tool *RefFinder* (http://www.leonxie.com/referencegene.php).

## Results

### Primer specificity and efficiency

Seven candidate reference genes were visualized as a single amplicon on a 1.5% agarose gel. Furthermore, gene-specific amplification of these genes was confirmed by a single peak in melting-curve analysis (S1 Fig). The correlation coefficient (R^2^) and PCR efficiency for each standard curve were shown in Table 1.

### *C*_*t*_ values of candidate reference genes

**Fig 1:**
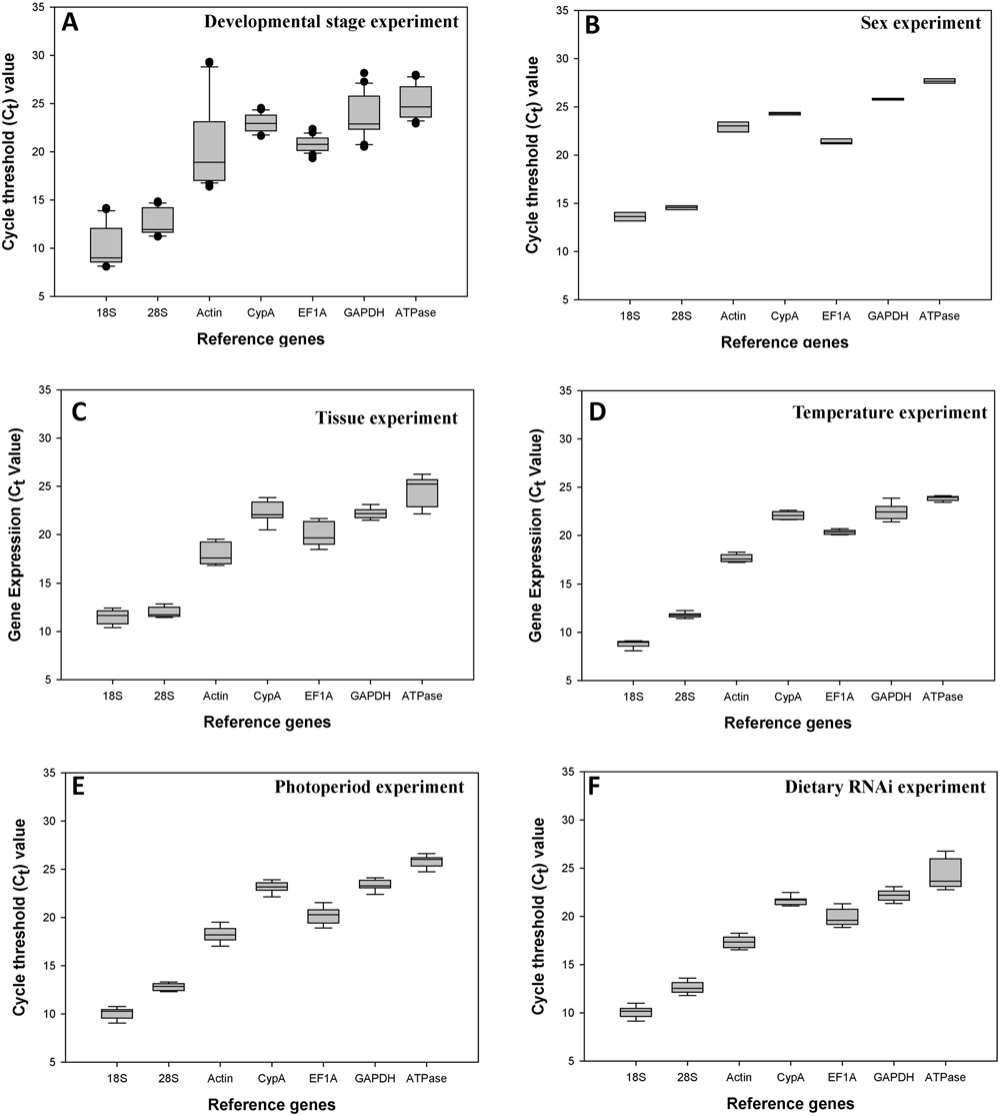
Expression profiles of the seven candidate reference genes in all six experiments in *Hippodamia convergens*. The dot indicates the maximum or minimum value of replicated samples, while whiskers indicate the standard error of the mean.

For the developmental stage experiment, the *C*_*t*_ values ranged from 10 to 25. *18S* and *28S* showed average *C*_*t*_ values below 15 cycles. *Actin* was less expressed in the egg stage with the *C*_*t*_ values above 28. *18S* and *ATPase* were the most and the least expressed reference gene, respectively (Fig 1A).

For the sex experiment, the *C*_*t*_ values ranged from 13 to 28. *18S* and *28S* showed average *C*_*t*_ values below 15 cycles. All of the seven candidate reference genes were equally expressed in both female and male adults. *18S* and *ATPase* were the most and the least expressed reference gene, respectively (Fig 1B).

For the tissue experiment, the *C*_*t*_ values ranged from 11 to 24. *18S* and *28S* showed average *C*_*t*_ values below 12 cycles. *Actin* showed average *C*_*t*_ values below 18. *18S* and *ATPase* were the most and the least expressed reference gene, respectively (Fig 1C).

For the temperature experiment, the *C*_*t*_ values ranged from 9 to 24. *18S* and *28S* showed average *C*_*t*_ values below 12 cycles. *Actin* showed average *C*_*t*_ values below 18. *EF1A*, *GAPDH*, and *CypA* showed average *C*_*t*_ values below 23. *18S* and *ATPase* were the most and the least expressed reference gene, respectively (Fig 1D).

For the photoperiod experiment, the *C*_*t*_ values ranged from 10 to 26. *18S*, *28S*, and *Actin* showed average *C*_*t*_ values below 18 cycles. *EF1A* showed the *C*_*t*_ values around 20, *GAPDH* and *CypA* were around 23. *18S* and *ATPase* were the most and the least expressed reference gene, respectively (Fig 1E).

For the dietary RNAi experiment, the *C*_*t*_ values ranged from 10 to 24. *18S*, *28S*, *Actin*, and *EF1A* showed average *C*_*t*_ values below 20 cycles, *GAPDH* and *CypA* were around 22. The expression of *ATPase* was significantly depressed in the *ATPase*-dsRNA supplied samples compared to the water and GUS-dsRNA fed controls. *18S* and *ATPase* were the most and the least expressed reference gene, respectively (Fig 1F).

### Stability of candidate reference genes under biotic conditions

*GeNorm* bases its ranking on the geometric mean of the standard deviation (SD) of each transformed gene set of pair combinations (M-value). The lower the M-value is, the higher the ranking. Under the impact of developmental stage, *CypA* and *EF1A* were co-ranked as the most stable genes. The overall order based on *geNorm* from the most stable to least stable reference gene was: *CypA* = *EF1A*, *28S*, *ATPase*, *18S*, *GAPDH*, *Actin* (Table 2). For the tissue experiment, *28S* and *GAPDH* were co-ranked as the most stable genes. The overall order based on *geNorm* from the most stable to least stable reference gene was: *28S* = *GAPDH*, *CypA*, *EF1A*, *ATPase*, *18S*, *Actin* (Table 2). For the sex experiment, *CypA* and *GAPDH* were co-ranked as the most stable genes. The overall order based on *geNorm* from the most stable to least stable reference gene was: *CypA* = *GAPDH*, *28S*, *EF1A*, *ATPase*, *18S*, *Actin* (Table 2).

**Table 2:** Stability of reference gene expression under biotic conditions.

Biotic conditions	Reference gene	*geNorm*		*Normfider*		*BestKeeper*		*ΔCt*	
		Stability	Rank	Stability	Rank	Stability	Rank	Stability	Rank
Stage	*28S*	0.92	2	0.57	2	1.13	3	1.48	1
	*CypA*	0.77	1	1.15	4	0.77	2	1.68	3
	*EF1A*	0.77	1	1.24	5	0.70	1	1.78	4
	*ATPase*	1.01	3	0.39	1	1.47	4	1.49	2
	*GAPDH*	1.35	5	0.91	3	1.92	6	1.84	5
	*18S*	1.15	4	1.33	6	1.82	5	1.85	6
	*Actin*	1.93	6	3.26	7	3.35	7	3.37	7
Tissue	*28S*	0.28	1	0.14	1	0.44	2	0.88	2
	*CypA*	0.49	2	0.46	2	0.82	4	0.95	3
	*EF1A*	0.54	3	0.71	3	0.97	6	1.07	4
	*ATPase*	0.79	4	1.33	5	1.29	7	1.48	6
	*GAPDH*	0.28	1	0.14	1	0.41	1	0.87	1
	*18S*	1.00	5	1.15	4	0.57	3	1.43	5
	*Actin*	1.19	6	1.54	6	0.90	5	1.67	7
Sex	*28S*	0.28	2	0.14	2	0.18	3	0.38	2
	*CypA*	0.24	1	0.28	3	0.15	2	0.44	3
	*EF1A*	0.33	3	0.38	4	0.26	5	0.50	4
	*ATPase*	0.35	4	0.41	5	0.22	4	0.51	5
	*GAPDH*	0.24	1	0.08	1	0.09	1	0.37	1
	*18S*	0.43	5	0.48	6	0.38	6	0.57	6
	*Actin*	0.48	6	0.53	7	0.44	7	0.60	7

A low stability value suggests a more stable gene by *NormFinder*. Under the impact of developmental stage, *ATPase* was the most stable gene. The overall order based on *NormFinder* from the most stable to least stable reference gene was: *ATPase*, *28S*, *GAPDH*, *CypA*, *EF1A*, *18S*, *Actin* (Table 2). For the tissue experiment, *28S* and *GAPDH* were co-ranked as the most stable genes. The overall order based on *NormFinder* from the most stable to least stable reference gene was: *28S* = *GAPDH*, *CypA*, *EF1A*, *18S*, *ATPase*, *Actin* (Table 2). For the sex experiment, *GAPDH* was the most stable gene. The overall order based on *NormFinder* from the most stable to least stable reference gene was: *GAPDH*, *28S*, *CypA*, *EF1A*, *ATPase*, *18S*, *Actin* (Table 2).

The stability of a gene is inversely proportional to the *BestKeeper* computed standard deviation (SD) value. Under the impact of developmental stage, *EF1A* was the most stable gene. The overall order based on *BestKeeper* from the most stable to least stable reference gene was: *EF1A*, *CypA*, *28S*, *ATPase*, *18S*, *GAPDH*, *Actin* (Table 2). For the tissue experiment, *GAPDH* was the most stable gene. The overall order based on *BestKeeper* from the most stable to least stable reference gene was: *GAPDH*, *28S*, *18S*, *CypA*, *Actin*, *EF1A*, *ATPase* (Table 2). For the sex experiment, *GAPDH* was the most stable gene. The overall order based on *BestKeeper* from the most stable to least stable reference gene was: *GAPDH*, *CypA*, *28S*, *ATPase*, *EF1A*, *18S*, *Actin* (Table 2).

The *ΔC*_*t*_ method relies on relative pair-wise comparisons. Using raw *C*_*t*_ values, the average SD of each gene set is inversely proportional to its stability. Under the impact of developmental stage, *28S* was the most stable gene. The overall order based on *ΔC*_*t*_ method from the most stable to least stable reference gene was: *28S*, *ATPase*, *CypA*, *EF1A*, *GAPDH*, *18S*, *Actin* (Table 2). For the tissue experiment, *GAPDH* was the most stable gene. The overall order based on *ΔC*_*t*_ method from the most stable to least stable reference gene was: *GAPDH*, *28S*, *CypA*, *EF1A*, *18S*, *ATPase*, *Actin* (Table 2). For the sex experiment, *GAPDH* was the most stable gene. The overall order based on *ΔC*_*t*_ method from the most stable to least stable reference gene was: *GAPDH*, *28S*, *CypA*, *EF1A*, *ATPase*, *18S*, *Actin* (Table 2).

**Fig 2:**
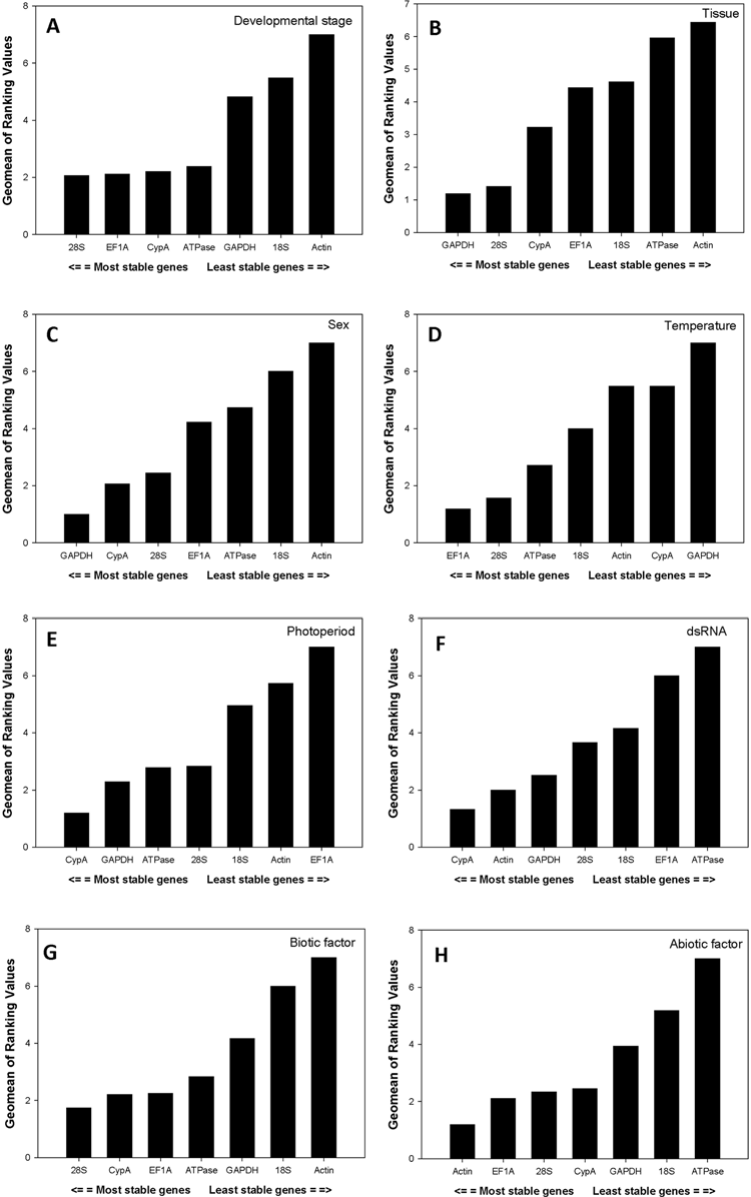
Stability of candidate reference genes expression under different treatment according to their stability value by *RefFinder*. A lower *Geomean* value indicates more stable expression.

Under the impact of developmental stage, according to *RefFinder*, which integrates the above-mentioned four software tools to compare and rank the candidates, the comprehensive ranking of candidate reference gene from the most to the least stable was: *28S*, *EF1A*, *CypA*, *ATPase*, *GAPDH*, *18S*, *Actin* (Fig 2A). For the tissue experiment, the comprehensive ranking of candidate reference gene from the most to the least stable was: *GAPDH*, *28S*, *CypA*, *EF1A*, *18S*, *ATPase*, *Actin* (Fig 2B). For the sex experiment, the overall ranking from the most to the least stable reference gene in both adult females and males was: *GAPDH*, *CypA*, *28*S, *EF1A*, *ATPase*, *18S*, *Actin* (Fig 2C). To summarize, the overall ranking from the most to the least stable reference gene under the biotic condition was: *28S*, *CypA*, *EF1A*, *ATPase*, *GAPDH*, *18S*, *Actin* (Fig 2G).

### Stability of candidate reference genes under abiotic conditions

Based on *GeNorm*, for the temperature experiment, *28S* and *EF1A* were co-ranked as the most stable genes. The overall order based on *geNorm* from the most stable to least stable reference gene was: *28S* = *EF1A*, *ATPase*, *18S*, *Actin*, *CypA*, *GAPDH* (Table 3). For the photoperiod experiment, *ATPase* and *GAPDH* were co-ranked as the most stable genes. The overall order based on *geNorm* from the most stable to least stable reference gene was: *ATPase* = *GAPDH*, *CypA*, *28S*, *Actin*, *EF1A*, *18S* (Table 3). For the dietary RNAi experiment, *Actin* and *GAPDH* were co-ranked as the most stable genes. The overall order based on *geNorm* from the most stable to least stable reference gene was: *Actin* = *GAPDH*, *CypA*, *28S*, *18S*, *EF1A*, *ATPase* (Table 3).

**Table 3:** Stability of reference gene expression under abiotic conditions.

Abiotic conditions	Reference gene	*geNorm*		*Normfider*		*BestKeeper*		*ΔCt*	
		Stability	Rank	Stability	Rank	Stability	Rank	Stability	Rank
Temperature	*28S*	0.19	1	0.15	3	0.17	1	0.38	2
	*CypA*	0.35	5	0.39	5	0.35	5	0.53	5
	*EF1A*	0.19	1	0.10	1	0.19	2	0.37	1
	*ATPase*	0.21	2	0.14	2	0.19	2	0.39	3
	*GAPDH*	0.50	6	0.83	7	0.59	6	0.87	7
	*18S*	0.22	3	0.18	4	0.26	3	0.41	4
	*Actin*	0.30	4	0.44	6	0.33	4	0.55	6
Photoperiod	*28S*	1.44	3	1.50	4	0.47	1	2.57	4
	*CypA*	1.30	2	0.66	2	1.77	5	2.35	2
	*EF1A*	2.25	5	3.51	6	1.53	4	3.73	6
	*ATPase*	1.15	1	0.78	3	1.50	3	2.39	3
	*GAPDH*	1.15	1	0.57	1	0.79	2	2.25	1
	*18S*	3.04	6	1.50	7	5.11	7	4.99	7
	*Actin*	1.87	4	1.75	5	2.54	6	2.96	5
	*28S*	0.43	5	0.48	6	0.38	6	0.57	6
	*CypA*	0.48	6	0.53	7	0.44	7	0.60	7
dsRNA	*28S*	0.45	3	0.42	3	0.46	5	0.65	3
	*CypA*	0.28	2	0.21	1	0.35	1	0.60	1
	*EF1A*	0.53	5	0.52	6	0.72	6	0.75	6
	*ATPase*	0.76	6	1.29	7	1.27	7	1.35	7
	*GAPDH*	0.25	1	0.44	4	0.44	2	0.68	5
	*18S*	0.48	4	0.48	5	0.45	3	0.68	4
	*Actin*	0.25	1	0.22	2	0.45	4	0.62	2

Based on *NormFinder*, for the temperature experiment, *EF1A* was the most stable gene. The overall order based on *NormFinder* from the most stable to least stable reference gene was: *EF1A*, *ATPase*, *28S*, *18S*, *CypA*, *Actin*, *GAPDH* (Table 3). For the photoperiod experiment, *GAPDH* was the most stable gene. The overall order based on *NormFinder* from the most stable to least stable reference gene was: *GAPDH*, *CypA*, *ATPase*, *28S*, *Actin*, *EF1A*, *18S* (Table 3). For the dietary RNAi experiment, *CypA* was the most stable gene. The overall order based on *NormFinder* from the most stable to least stable reference gene was: *CypA*, *Actin*, *28S*, *GAPDH*, *18S*, *EF1A*, *ATPase* (Table 3).

Based on *BestKeeper*, for the temperature experiment, *28S* was the most stable gene. The overall order based on *BestKeeper* from the most stable to least stable reference gene was: *28S*, *EF1A* = *ATPase*, *18S*, *Actin*, *CypA*, *GAPDH* (Table 3). For the photoperiod experiment, *28S* was the most stable gene. The overall order based on *BestKeeper* from the most stable to least stable reference gene was: *28S*, *GAPDH*, *ATPase*, *EF1A*, *CypA*, *Actin*, *18S* (Table 3). For the dietary RNAi experiment, *CypA* was the most stable gene. The overall order based on *BestKeeper* from the most stable to least stable reference gene was: *CypA*, *GAPDH*, *18S*, *Actin*, *28S*, *EF1A*, *ATPase* (Table 3).

Based on *ΔC*_*t*_ method, for the temperature experiment, *EF1A* was the most stable gene. The overall order based on *ΔC*_*t*_ method from the most stable to least stable reference gene was: *EF1A*, *28S*, *ATPase*, *18S*, *CypA*, *Actin*, *GAPDH* (Table 3). For the photoperiod experiment, *GAPDH* was the most stable gene. The overall order based on *ΔC*_*t*_ method from the most stable to least stable reference gene was: *GAPDH*, *CypA*, *ATPase*, *28S*, *Actin*, *EF1A*, *18S* (Table 3). For the dietary RNAi experiment, *CypA* was the most stable gene. The overall order based on *ΔC*_*t*_ method from the most stable to least stable reference gene was: *CypA*, *Actin*, *28S*, *18S*, *GAPDH*, *EF1A*, *ATPase* (Table 3).

Based on *RefFinder*, for the temperature experiment, the comprehensive ranking of candidate reference gene from the most to the least stable was: *EF1A*, *28S*, *ATPase*, *18S*, *Actin*, *CypA*, *GAPDH* (Fig 2D). For the photoperiod experiment, the overall ranking from the most to the least stable reference gene under the photoperiod stress was: *CypA*, *GAPDH*, *ATPase*, *28S*, *18S*, *Actin*, *EF1A* (Fig 2E). For the dietary RNAi experiment, the overall ranking from the most to the least stable reference gene under the dsRNA stress was: *CypA*, *Actin*, *GAPDH*, *28S*, *18S*, *EF1A*, *ATPase* (Fig 2F). To summarize, the overall ranking from the most to the least stable reference gene under the abiotic condition was: *Actin*, *EF1A*, *28S*, *CypA*, *GAPDH*, *18S*, *ATPase* (Fig 2H).

### Quantitative analysis of candidate reference genes based on *geNorm*

**Fig 3:**
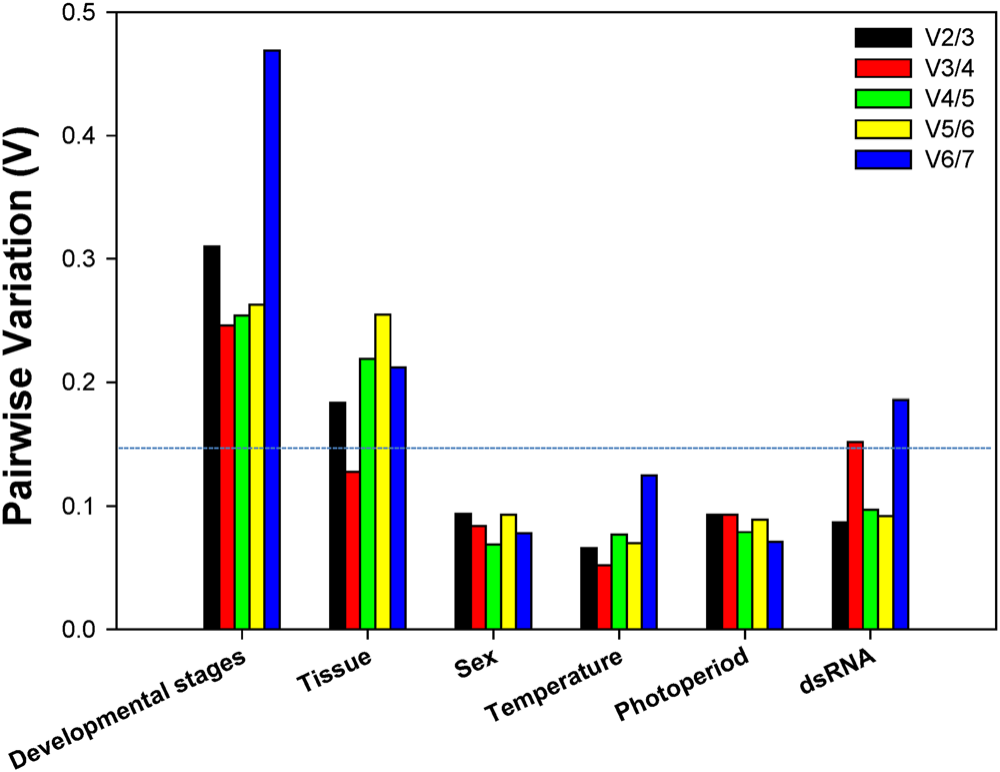
Pairwise variation (V) analysis of the candidate reference genes. The *geNorm* first calculates an expression stability value (M) for each gene and then compares the pair-wise variation (V) of this gene with the others. A threshold of V<0.15 was suggested for valid normalization. Starting with two genes, the software sequentially adds another gene and recalculates the normalization factor ratio. If the added gene does not increase the normalization factor ratio above the proposed 0.15 cut-off value, then the original pair of genes is enough for normalization. However, if the new ratio is above 0.15, then more genes should be included [1].

To decide the minimal number of reference genes mandatory for normalization, the V-value was computed by *geNorm*. Beginning with two genes, the software continuously adds another gene and recalculates the normalization factor ratio. If the added gene does not elevate the normalization factor ratio over the proposed 0.15 cut-off value, then the starting pair of genes is sufficient for the normalization. Otherwise, more genes should be incorporated. However, if the new ratio is above 0.15, then more genes should be included. For the developmental stage experiment, although none of the V-value was less than 0.15, the lowest value appeared at V3/4, suggesting that three reference genes were desirable for reliable normalization throughout developmental stages (Fig 3). For the tissue experiment, the first V-value < 0.15 emerged at V3/4, suggesting that three reference genes were required for reliable normalization in different tissue types (Fig 3). For the sex, temperature, photoperiod, and dietary RNAi experiments, the first V-value < 0.15 showed at V2/3, recommending that two reference genes were sufficient for reliable normalization (Fig 3).

## Discussion

RT-qPCR quantification requires a comprehensive normalization by reference genes to counteract confounding variation in experimental data. Generally considered to be expressed in all cell types of an organism at a constant level to maintain basic cellular functions, reference genes have been widely adopted as internal controls for various analyses involving gene quantification, including Western blot, Northern blot, and RT-qPCR. Each candidate reference gene, however, should be evaluated under specific experimental conditions for gene profiling to ensure a constant level of expression [23]. Our results demonstrate that the best suited reference genes can be different in response to different biotic and abiotic conditions. This is consistent with previous reports showing that reference genes are differentially expressed under specific experimental conditions in the sweet potato whitefly, *Bemisia tabaci* (Hemiptera: Aleyrodidae), diamondback moth, *Plutella xylostella* (Lepidoptera: Plutellidae), brown planthopper, *Nilaparvata lugens* (Hemiptera: Delphacidae), beet armyworm, *Spodoptera exigua* (Hübner) (Lepidoptera: Noctuidae), oriental leafworm moth, *Spodoptera litura* (Lepidoptera: Noctuidae), oriental fruit fly, *Bactrocera dorsalis* (Diptera: Tephritidae), pea aphid, *Acyrthosiphon pisum* (Harris) (Hemiptera, Aphidiae), and Colorado potato beetle, *Leptinotarsa decemlineata* (Say) (Coleoptera: Chrysomelidae) [5, 6, 24–29]. Even under the same insecticide treatment, different classes of insecticides warranted different sets of reference genes to normalize target gene expression in *B*. *tabaci*, indicating the necessity for custom reference gene selection [30].

The large body of recent works clearly suggested that there are no "universal" reference genes that are stably expressed and applicable for all cell and tissue types and various experimental conditions [5, 6, 27–29]. For example, as a major component of the protein scaffold which supports the cell and determines its shape, *Actin* has been widely regarded as the universal reference gene and used extensively as the internal reference without any validation. For *H*. *convergens*, however, *Actin* was the least stable reference gene under different biotic conditions (developmental stage, tissue, and sex). Recently, Lin and Redies [31] compared histological expression profile of *GAPDH* and *Actin* in the developing chicken embryo. Neither *GAPDH* nor *Actin* was expressed in all cell types or tissues at high levels, and in some organs, these two reference genes exhibited partially complementary expression patterns. Specifically, *Actin* is highly expressed in the gizzard, but it was virtually non-existent in cardiac muscle cells.

There has been ongoing discussion about the optimal number of reference genes required for RT-qPCR analysis. Previously, gene expression studies have predominantly used a single endogenous control, however, this will profoundly influence the statistical outcome and may lead to inaccurate data interpretation [32] or it is simply insufficient to normalize the expression of target genes [33]. To avoid biased normalization, more and more researchers have moved away from a single endogenous control and started to embrace the idea of using multiple reference genes to analyze gene expression [5, 6, 27–29]. Determination of the optimal number of reference genes, however, has always being a trade-off between accuracy and practicality. A minimum of three most stably expressed reference genes is recommended by Thomas and colleagues [34]. In this study, two reference genes are sufficient to normalize the expression and provide a more conservative estimation of target gene expression under abiotic conditions, including temperature, photoperiod, and dietary RNAi. In contrast, three stable reference genes are generally required under biotic conditions, including developmental stage and tissue.

This is the first study to investigate candidate reference genes for gene expression analyses in the predatory species *H*. *convergens*. Based on a comprehensive analysis integrating five commonly used analytical methods to compare and rank the candidate reference genes under an array of biotic (developmental stage, tissue, and sex) and abiotic (temperature, photoperiod, and dietary RNAi) conditions, a suite of candidate reference genes were specifically recommended for each experimental condition. Among them, *28S*, *EF1A*, and *CypA* were the best reference genes across different development stage; *GAPDH*, *28S*, and *CypA* were most stable in different tissues. *GAPDH* and *CypA* were most stable in female and male adults and photoperiod conditions, *28S* and *EF1A* were most stable under a range of temperatures, *Actin* and *CypA* were most stable under dietary RNAi condition. This work is the initial first step to establish a standardized RT-qPCR analysis in *H*. *convergens*. Additionally, this study lays a foundation for the functional genomics research in *H*. *convergens*, and sheds light on the ecological risk assessment of RNAi-based biopesticides on this non-target biological control agent.

## Supporting Information

S1 Fig Melting curve of the seven candidate reference genes. (TIFF)

S1 Table Degenerate primers used for RT-qPCR. (DOCX)

S2 Table The sequencing results of these five genes including *Actin*, *EF1A*, *GAPDH*, *CypA*, *ATPase* using the degenerate primers. (DOCX)
