# Supplementary figures and images for: A Comprehensive Selection of Reference Genes for RT-qPCR Analysis in a Predatory Lady Beetle, Hippodamia convergens (Coleoptera: Coccinellidae)
Source: PLoS One. 2015 Apr 27;10(4):e0125868. doi: 10.1371/journal.pone.0125868 (PMC4411045; doi:10.1371/journal.pone.0125868)

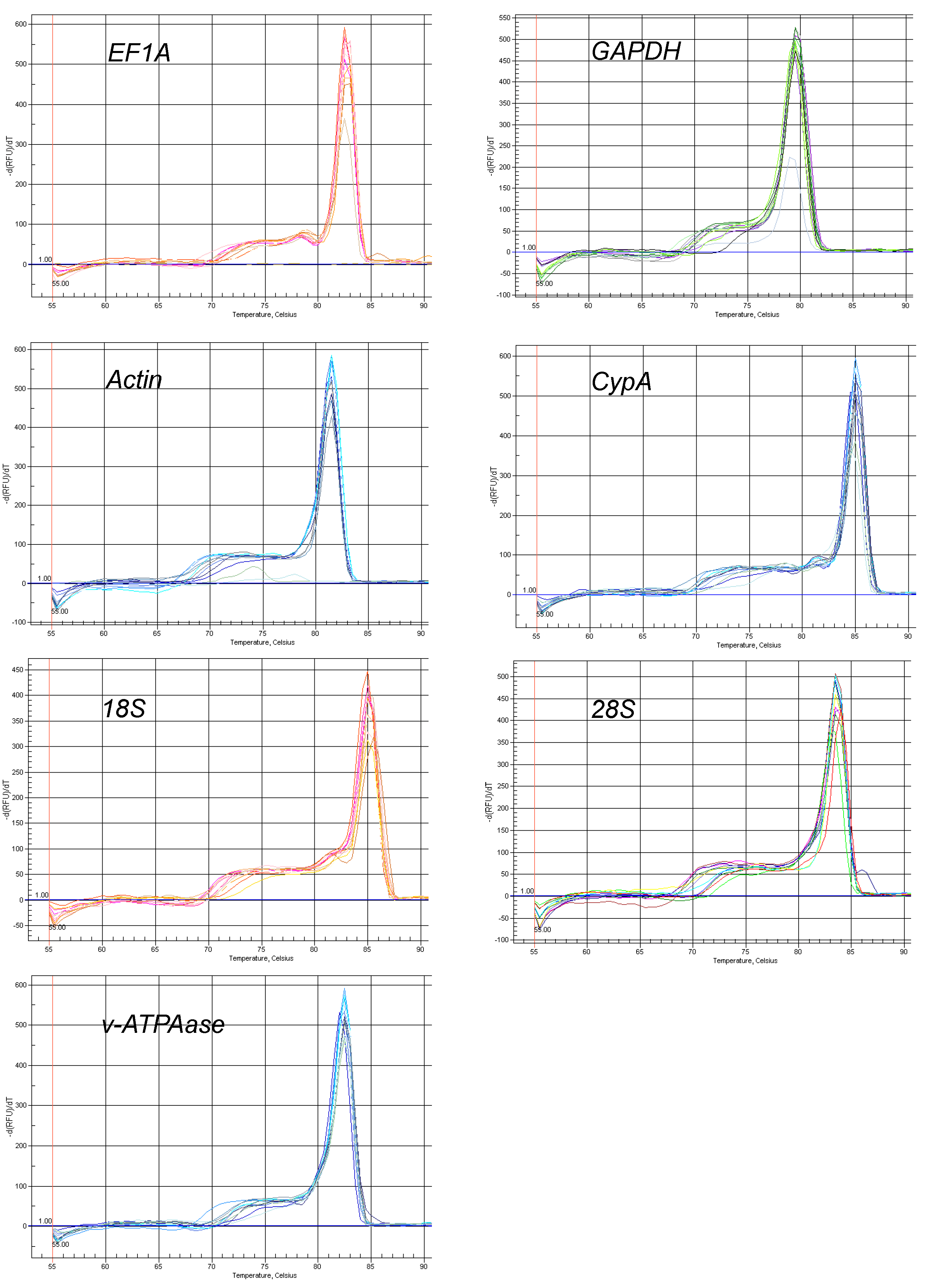

Supplement: S1 Fig — (TIFF) [file pone.0125868.s001.tiff]
