# Supplementary material for: A Comprehensive Selection of Reference Genes for RT-qPCR Analysis in a Predatory Lady Beetle, Hippodamia convergens (Coleoptera: Coccinellidae)
Source: PLoS One. 2015 Apr 27;10(4):e0125868. doi: 10.1371/journal.pone.0125868 (PMC4411045; doi:10.1371/journal.pone.0125868)
Supplement: S1 Table — (DOCX) [file pone.0125868.s002.docx]

**S1 Table. Degenerate primers used for qPCR**

| Gene | Primer sequences (5’-3’) |
| --- | --- |
| *ATPase* | F: AGATGTCCGGATCNgctatgtacga |
|  | R: ACGAGCAGCCacaggcatgtt |
| *GAPDH* | F: AACGCCTCCTGCACCacnaaytgyyt |
|  | R: CGGGCACGCGGaangccatncc |
| *Actin* | F: CGACATGGAAAAGATCtggcaycayac |
|  | R: TCGGTCAGCGATACCAggrtacatngt |
| *CypA* | F: TGTGCACAGGTGAAAAAGGTttyggntayaa |
|  | R: CCTTCCACCACGTTACCGaanacnacrtg |
| *EF1A* | F: TCGACATCGCTCTGTGGaarttygarac |
|  | R: GTACCGATACCACCGATTTTGtanacrtcytg |
