# Supplementary material for: A Comprehensive Selection of Reference Genes for RT-qPCR Analysis in a Predatory Lady Beetle, Hippodamia convergens (Coleoptera: Coccinellidae)
Source: PLoS One. 2015 Apr 27;10(4):e0125868. doi: 10.1371/journal.pone.0125868 (PMC4411045; doi:10.1371/journal.pone.0125868)
Supplement: S2 Table — (DOCX) [file pone.0125868.s003.docx]

**S2 Table. The sequencing results of these five genes including *Actin*, *EF1A*, *GAPDH*, *CypA*, *ATPase* using the degenerate primers**

>*CypA*

CCTTCCACCACGTTACCGAAGACGACGT

GCCTGTTGTCCAGCCAGGAGGTTTTCACGGTGGTGATGAAGAACTGCGATCCGTTGGTGT

CGGGTCCGGCGTTCGCCATGGACATCACTCCGGCGCCGGTGTGTTTCAGGGTGAAGTTCT

CGTCGGGGAATTTGCTGCCGTAGATGGACTTGCCCCCGGTGCCGTTGTGGTTGGTGAAGT

CACCACCTTGGCACATGAAGTTGGGGATGACGCGGTGGAAGGTGGATCCCTTATATCCAA

AACCTTTTTCACCTGTGCACA

>*EF1A*

GTACCGATACCACCGATTTTGTACACGTCTTGGAGTGGAAGACGGAGGGGTTTCTCAGTT

GGACGAGATGGAGGAAGAATGGCGTCAAGAGCTTCAATCAGGCATTTACCGTCAGCCTTT

CCTTCTTTACGTTCAATTGCCCATCCCTTGAACCATGGCATTTTGGTGGAAGGCTCCAAC

ATGTTGTCTCCGTGCCATCCTGAGATGGGTACGAATGCAACGGCTGCTGGGTTGTAACCG

ATCTTCTTGATGTACGAAGATACTTCCTTCTTGATTTCCTCGAAACGAGCTTCACTGTAT

GGTGGTTCGGTGGAGTCCATCTTGTTGACTCCAACGATGAGTTGTTTCACACCCAAGGTG

AAGGCGAGCAGAGCGTGCTCACGAGTTTGTCCGTTCTTTGAGATACCAGCCTCGAATTCA

CCAGTACCGGCAGCAACAATCAGTACAGCACAATCCGCTTGGGATGTTCCGGTGATCATG

TTCTTGATGAAATCCCTGTGTCCAGGGGCATCAATGATGGTTACGTAGTATTTGGAGGTC

TCAAACTTCCACAGAGCGATGTCGA

>*GAPDH*

AACGCCTCCTGCACCACGAATTGCCTGGCCCCAT

TGGCCAAGGTGATCCATGACAAATTTGGAATTGTTGAAGGTCTGATGACTACTGTCCATG

CTACAACTGCCACTCAGAAGACTGTTGACGGTCCGTCAGGAAAATTGTGGCGTGATGGTC

GTGGTGCTGCTCAAAACATCATTCCAGCCTCCACCGGAGCAGCCAAGGCTGTAACCAAGG

TCATCAAGTCCCTGGAAGGAAAGTTGACTGGGATGGCCTTCCGCGTGCCCG

>*Actin*

TCAGCGATACCAGGGTACATAGTGGTACCTCCTGAGAGTACAGTGTTGGCGTATAAGTCCTTACGGATGTCCACGTCACACTTCATGATGGAGTTGTAGACGGTCTCGTGGATTCCGCAAGATTCCATACCCAAGAAGGAAGGCTGGAAGAGGGCTTCAGGGCAACGGAACCTCTCGTTACCAATGGTGATGACCTGACCGTCGGGAAGTTCGTAGGACTTCTCCAAAGAGGTGGAGGCGGCAGCGGTGGCCATTTCTTGCTCGAAGTCGAGGGCGACATAGCAGAGTTTTTCCTTGATGTCACGTACAATTTCCCTTTCAGCGGTGGTTGTGAAAGAGTAACCCCTTTCGGTGAGGATCTTCATGAGGTAGTCGGTCAAGTCACGACCAGCCAAGTCCAAACGTAGGATGGCGTGGGGAAGAGCGTAACCTTCGTAGATTGGTACAGTGTGGGACACACCATCTCCAGAATCCAACACGATACCAGTGGTACGACCGGAAGCGTACAATGAAAGTACAGCTTGAATAGCGACGTACATGGCTGGGGTGTTGAAGGTTTCAAACATGATTTGGGTCATCTTCTCCCTGTTAGCTTTGGGGTTCAAGGGAGCTTCAGTCAAGAGGACGGGGTGTTCTTCTGGAGCTACACGAAGTTCGTTGTAGAAGGTATGATGCCAGATCTTTTCCATGTCG

>*ATPase*

ATGTGCGGATCGGCTATGTACGAGCTTGTGAGAGTTGGATACTTTGAATTGGTGGGTGAAATTATTCGTCTTGAAGGTGACATGGCAACAATTCAGGTATACGAAGAAACTTCTGGTGTAACGGTTGGAGATCCTGTTCTACGTACTGGTAAACCATTGTCTGTAGAATTAGGTCCTGGTATAATGGGTTCAATTTTTGATGGTATCCAGCGTCCTTTGAAAGATATCAATGTTCTCACAGAAAGTATCTACATTCCCAAAGGTATCAACGTGCCTTGCTTGTCTAGAACTGCTAAGTGGGACTTCAATCCTACCAACATTAAAATGGGATCTCACTTAACGGGTGGAGATATTTACGGTATTGTCCATGAAAATACTTTGGTAAAACAAAAACTCATGTTGCCGCCAAAGTCGAAAGGTACAGTTACCTATATTGCAGAACCAGGAAGTTACACTGTCGATGATGTTGTTTTGGAAACTGAATTCGATGGCGAACGCACAAAATACACTATGTTGCAAGTTTGGCCAGTCCGTCAGCCGCGTCCAGTGAGCGAAAAATTGCCAGCAAATCATCCTCTTCTTACTGGACAGAGAGTTTTAGATTCTCTCTTCCCATGTGTACAAGGGGGTACCACTGCCATCCCTGGTGCCTTCGGTTGTGGAAAAACTGTAATCTCTCAATCTCTATCCAAATATTCTAATTCTGATGTCATCATCTATGTAGGATGTGGAGAAAGAGGTAACGAAATGTCTGAAGTACTTCGTGACTTCCCCGAACTGACAGTCGAGATCGAAGGCCAGACTGAATCCATCATGAAACGTACCGCTCTTGTAGCGAACACGTCCAACATGCCTGTCGCCGCTCGT
